# What Do Antibody Studies Tell Us about Viral Infections?

**DOI:** 10.3390/pathogens11050560

**Published:** 2022-05-10

**Authors:** Philipp A. Ilinykh, Kai Huang

**Affiliations:** 1 Department of Pathology, University of Texas Medical Branch, Galveston, TX 77555, USA; kahuang@utmb.edu; 2 Galveston National Laboratory, University of Texas Medical Branch, Galveston, TX 77550, USA

Humoral immunity is an important body defense system against virus infection and is correlated to patient health status. Antibody response is a key factor in controlling virus replication. During infection, viruses induce the production of antibodies, which differ in their isotype, neutralization capacity and breadth, recognition of surface versus internal viral proteins, and epitope specificity. These and other yet to be identified factors determine the role of antibodies in virus clearance through the direct neutralization and Fc effector functions, such as antibody-dependent cellular cytotoxicity [1]. However, certain features of the antibody response, such as antibody-dependent enhancement of infection [2] or increased inflammation resulting from the deposition of immune complexes [3], can create “adverse effects” to exacerbate infection. Adding to the complexity of interaction between viruses and host immune systems, some viruses have exploited multiple mechanisms to compromise antibody production, which helps them to overcome the resistance of host organisms and establish infection. This phenomenon often contributes to the differences in magnitude and longevity of the humoral response to natural infections, in comparison with vaccines [4]. Despite recent advancements in the characterization of monoclonal antibody responses to a number of human pathogens, including human immunodeficiency virus 1 [5], influenza virus [6], dengue virus [7], chikungunya virus [8], rabies virus [9], paramyxoviruses [10], poxviruses [11], hantaviruses [12], filoviruses [13], and coronaviruses [14], critical knowledge gaps still exist. In particular, many viral and host factors that determine the dynamics of antibody response and their role in pathogenesis, as well as the mechanisms of antiviral and proviral antibody effects, remain undefined. Undoubtedly, this information will be vital to guide the design of vaccines and therapeutic strategies based on passive immunization.

The Special Issue “Characterization of Antibody Responses to Virus Infections in Humans” has gathered nine publications, including seven original articles and two reviews, that emphasize the need for better understanding of biological aspects of humoral immune response to different viral pathogens.

The varicella-zoster virus (VZV) belongs to the *Herpesviridae* family and is the causative agent of varicella (chickenpox) and herpes zoster (shingles). After primary replication in the upper respiratory tract, VZV is transported via the bloodstream to the skin sites, causing a widely distributed vesicular rash. VZV can further reach ganglia by axonal transport and establish a latent infection in the nervous system. In case of infection reactivation, the virus is transported down the nerve to the correlating dermatome, which results in zoster. Due to VZV neurotropism, the infection can provoke long-lasting postherpetic neuralgia, especially in elderly and immunocompromised individuals [15,16]. Availability of accurate methods for serodiagnostics of VZV-specific antibodies is needed for the timely treatment of clinical cases, implementation of quarantine measures, vaccination effectiveness control and routine epidemiological surveillance of VZV. As an alternative to commercial kits that are not evenly distributed worldwide, Kombe et al. [17] developed the highly sensitive diagnostic approach based on the chemiluminescent immunoassay, which can detect very low IgA, IgG and IgM titers against VZV-gE envelope glycoprotein in patients at the early stage of infection.

Influenza A viruses (IAV) constitute a large group of pathogens with high relevance for public health. IAVs have been shown to infect humans, pigs, horses, dogs, cats and sea mammals [18,19,20,21,22,23]. Wild waterfowl serve as a natural reservoir for the vast majority of IAV serotypes. In general, human IAVs cause seasonal flu outbreaks worldwide, with mild-to-severe respiratory symptoms. However, due to the segmented nature of the IAV genome, new viruses emerge as a result of genome reassortment in humans and animals. Given the lack of immunity to such viruses in the human population, these new variants have the potential to cause a pandemic with a high case-fatality ratio [18]. In addition, multiple cases of human infection with avian IAV, predominately the H5 subtype, have been described [24,25,26] since the first documented outbreak in Hong Kong in 1997 [27,28,29]. In severe cases, the infection is characterized by excessive lung inflammation resulting from the virus-induced cytokine storm, and can often be fatal [30,31]. Therefore, the serosurveillance studies in ‘hot areas’, such as South-East Asia, are critical to track the circulation, emergence and evolution of avian IAV to inform outbreak preparedness and response measures. Ilyicheva et al. [32] analyzed serum samples from Vietnamese residents and reported the detection of neutralizing antibodies to H5 avian IAV isolated in Vietnam and Russia in 2017–2018. These findings suggest an ongoing adaptation of the rapidly evolving H5 viruses to human hosts.

The most recent pandemic of viral disease has been caused by severe acute respiratory syndrome coronavirus-2 (SARS-CoV-2), a zoonotic pathogen that barely requires a special introduction nowadays. The World Health Organization declared the COVID-19 outbreak a Public Health Emergency of International Concern on 30 January 2020, and a pandemic on 11 March 2020. It spread rapidly around the world, causing more than 511 million cases and 6.2 million deaths as of 3 May 2022 [33]. The early clinical studies of COVID-19 in China revealed important characteristics of the disease [34,35]. The patients had pneumonia, and, in severe cases, developed acute respiratory distress syndrome and required oxygen therapy in intensive care units. Other common complications included acute cardiac injury and secondary infection. The leucopenia, lymphopenia and high serum levels of proinflammatory cytokines were identified as markers of disease severity. Since the last quarter of 2020, variant viruses have emerged in many parts of the world because of the high burden of infection and the adaptation of SARS-CoV-2 to human cells under immune pressure [36,37].

In this Special Issue, five different publications [38,39,40,41,42] have characterized the antibody response at population and molecular levels, contributing to a broader picture of SARS-CoV-2 epidemiology. Xiao et al. [38] conducted a large-scale screening of serum samples in the Guangdong province, China, between March and June 2020. The overall prevalence of virus-specific antibodies was low soon after the emergence of COVID-19 in Guangdong, suggesting an urgent need for vaccination to increase population immunity to SARS-CoV-2. Another study by Xu et al. [39] revealed a 4.5-fold increase in SARS-CoV-2 seroprevalence from Fall 2020 to February 2021 in the population of Western Pennsylvania, USA, which was shown to be driven both by infection and vaccination. Kazachinskaia et al. [40] analyzed the blood samples of COVID-19 patients in Novosibirsk city, Russia, and observed cross-reactivity of antibodies to SARS-CoV (2002) proteins. Additionally, their results suggested that high neutralization titer is not necessarily predictive of the infection survival. Huang et al. [41] presented the kinetics study of viral load, humoral immune response and the cytokine profile in a hospital patient cohort (January–March 2020) and were able to correlate these parameters with the disease severity during the initial outbreak in Taiwan. Another work from Germany by Heidepriem et al. [42] provided longitudinal characterization of the antibody response to SARS-CoV-2. The linear epitopes in viral proteome and specific glycan structures, targeted by antibodies from COVID-19 patients with moderate and mild disease, were identified. 

Filoviruses represent yet another group of pathogens with high relevance for global health and include one of the deadliest human pathogens known so far, Ebola (EBOV) and Marburg viruses. These and other members of the *Filoviridae* family can cause a severe disease, which is often accompanied by haemorrhagic manifestations and systemic multiorgan dysfunction, with fatality rates reaching as high as 90% [43]. Human outbreaks generally result from spillover events from infected animals, including bats and non-human primates [44]. The development of infection is believed to result from deep suppression of the host immune system and dysregulation of both innate and adaptive arms of immunity by filoviruses. In worst cases, the rapid disease progression culminates in the death of an infected individual in 1–2 weeks after the onset of symptoms. The largest epidemic of filovirus-induced disease occurred in 2013–2016 in West Africa and claimed the lives of 11,310 out of 28,616 people infected [45]. 

The filovirus glycoprotein (GP) is the sole envelope viral protein responsible for cell entry; hence, it serves as the primary target for antibody-based therapies and vaccine design efforts. Currently, monoclonal antibody (mAb) therapy has been shown to be the most effective treatment of filoviral infections after the onset of symptoms [46]. In this Special Issue, two comprehensive review papers by Hargreaves et al. [47] and Yu and Saphire [48] summarize the recent advancements in the characterization of neutralizing antibody responses against EBOV and other filoviruses. The authors discuss the role of epitope specificity and Fc effector functions in antiviral mechanisms employed by the most promising antibodies, the correlation of these parameters with in vivo protection by individual mAbs and mAb cocktails, the structural basis for cross-reactivity to ebolavirus species and the strategies to avoid viral escape from neutralizing antibodies.

In conclusion, we believe that this Special Issue underlines the importance of multi-level analysis of antibody responses in the context of virus infections. The data presented here contribute to a better understanding of epidemiological and molecular aspects of infectious diseases caused by publicly relevant viral pathogens, such as VZV, IAV, SARS-CoV-2 and filoviruses. We hope that this Special Issue will stimulate future studies on humoral immune response to inform the development of countermeasures against life-threatening viral infections.
